# Multimed: An Integrated, Multi-Application Platform for the Real-Time Recording and Sub-Millisecond Processing of Biosignals

**DOI:** 10.3390/s18072099

**Published:** 2018-06-30

**Authors:** Antoine Pirog, Yannick Bornat, Romain Perrier, Matthieu Raoux, Manon Jaffredo, Adam Quotb, Jochen Lang, Noëlle Lewis, Sylvie Renaud

**Affiliations:** 1 Laboratoire de l’Intégration du Matériau au Système (IMS), University of Bordeaux, Bordeaux INP, CNRS UMR 5218, F-33400 Talence, France; antoine.pirog@bordeaux-inp.fr (A.P.); yannick.bornat@ims-bordeaux.fr (Y.B.); noelle.lewis@ims-bordeaux.fr (N.L.); 2 Signalisation et physiopathologie cardiovasculaire, INSERM S-1180, University of Paris Sud, F-92296 Châtenay-Malabry, France; romain.perrier@u-psud.fr; 3 Institut de Chimie et Biologie des Membranes et des Nano-objets (CBMN), University of Bordeaux, CNRS UMR 5248, F-33600 Pessac, France; m.raoux@cbmn.u-bordeaux.fr (M.R.); manon.jaffredo@u-bordeaux.fr (M.J.); j.lang@cbmn.u-bordeaux.fr (J.L.); 4 Laboratoire d’Analyse et d’Architecture des Systèmes (LAAS), Federal University of Toulouse Midi-Pyrénées, CNRS UMR 8001, F-31031 Toulouse, France; adam.quotb@enseeiht.fr

**Keywords:** biosignal processing, electrophysiology, FPGA, multi-application, multi-channel, neural recording, pancreatic islet recording, real-time

## Abstract

Enhanced understanding and control of electrophysiology mechanisms are increasingly being hailed as key knowledge in the fields of modern biology and medicine. As more and more excitable cell mechanics are being investigated and exploited, the need for flexible electrophysiology setups becomes apparent. With that aim, we designed Multimed, which is a versatile hardware platform for the real-time recording and processing of biosignals. Digital processing in Multimed is an arrangement of generic processing units from a custom library. These can freely be rearranged to match the needs of the application. Embedded onto a Field Programmable Gate Array (FPGA), these modules utilize full-hardware signal processing to lower processing latency. It achieves constant latency, and sub-millisecond processing and decision-making on 64 channels. The FPGA core processing unit makes Multimed suitable as either a reconfigurable electrophysiology system or a prototyping platform for VLSI implantable medical devices. It is specifically designed for open- and closed-loop experiments and provides consistent feedback rules, well within biological microseconds timeframes. This paper presents the specifications and architecture of the Multimed system, then details the biosignal processing algorithms and their digital implementation. Finally, three applications utilizing Multimed in neuroscience and diabetes research are described. They demonstrate the system’s configurability, its multi-channel, real-time processing, and its feedback control capabilities.

## 1. Introduction

The growing number of technological advances in electronics and materials has enabled many innovative approaches in biology and medicine to interact with excitable cells and tissues. Information communicated by organs, tissues, and cells about their activity are recorded through biophysical sensors and produce information regarding body health. Electronics captures this information, either invasively or non-invasively, measuring the electrophysiological properties and responses of biological tissue. This ability to observe and analyze electrical signals emitted by networks of excitable cells has created new perspectives in fundamental research, expanding our knowledge of communication between cells and physiological responses to drugs. Likewise, in medical research, this knowledge provides new solutions to restore body functions using neuroprosthetics or artificial organs, which rely on the ability of electrical signals to also stimulate excitable tissues via physiological pathways.

The ability to interact with living tissues relies on electronic devices, ranging from recording electrodes and recording front-ends to signal processing systems and stimulators. While medical studies demand more emphasis on system power cost, safety, and ergonomics, there is a strong need for versatility in fundamental research. The variety in measurement paradigms calls for multi-application electrophysiology platforms with easy-to-change configurations and algorithms, as well as real-time processing and feedback control features. These platforms need to adapt to various tissues, as measured on different scales: signals may vary with either the material’s origin (human, primate, rodent, etc.), type of excitable tissue (brain, muscle, cardiac, or pancreatic cells), and the recording paradigm (in vitro, ex vivo, or in vivo) [1].

This paper presents Multimed, which is a versatile embeddable full-hardware platform for real-time recording, processing, and control of biosignals. Multimed, designed over the course of successive projects and in close collaboration with biologists and clinicians, is suitable for multiple applications.

**Figure 1:**
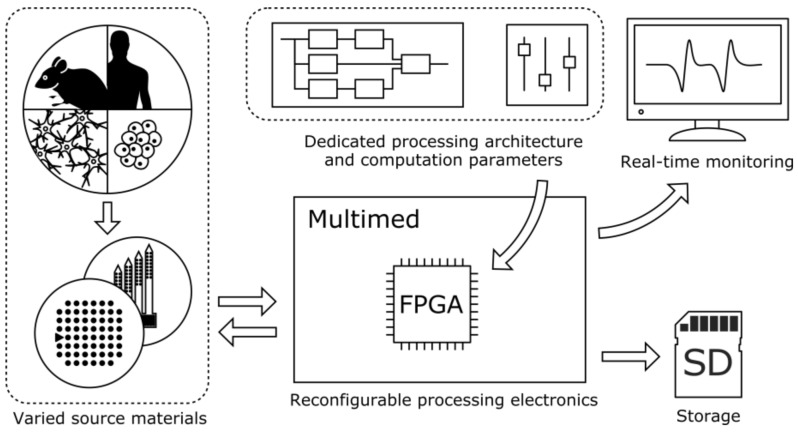
Multimed and its interfaces. From left to right: User parameters are fed into the system, which is connected to the measured and/or stimulated cell culture. The board then processes the data using a Field Programmable Gate Array (FPGA), which displays measurements on an external screen and records unprocessed and processed data to SD cards.

As depicted on Figure 1, Multimed is a system that is capable of acquiring and processing signals from various source materials, with a reconfigurable processing architecture taking advantage of an FPGA (Field Programmable Gate Array) chip. Recorded signals can then be displayed in real-time, stored, or utilized to generate feedback stimulation triggers. Overall, it may be used as a standalone analysis station for fundamental research or as a prototyping architecture for implantable prosthetics.

Signals from biological material are electrical readings of ionic fluxes at the plasma membrane captured by micro- or macro-electrodes. Signals include electrical signatures that are generated by the electrophysiology of a cell or clusters of cells, including the widely-described Action Potentials [2], a fast-pulsing activity where spectral components range above approximately 200 Hz, generated by a single cell. Some cells also show bursting activity, characterized by sequences of fast AP firing (>10 Hz) and silent periods. In a cell network, these signatures propagate via junctions (synapses or gap junctions) and trigger synchronized activity, known as network bursting. Clusters of depolarizing cells also generate Local Field Potentials (LFPs) [3], a biphasic oscillation with spectral components ranging below 100 Hz. On a larger scale, micro-organs, such as the pancreatic islets, also called islets of Langerhans, which are composed of many (hundreds to thousands of) excitable cells, display continuous oscillations, reflecting its syncytial behavior and constituting a biomarker of cell coupling. These Slow Potentials (SPs) have spectral components below 1 Hz [4].

Decoding information from these signals requires various forms of analog signal conditioning (amplification and filtering). Then, digital processing with statistical, frequency, or temporal analysis is necessary to extract features and produce relevant metrics. For the latter, adaptive information decoding is essential to take into account variations in signal and electrode properties, particularly for chronic recordings [5].

## 2. System Specifications

In this section, a description of our hardware platform is given. It includes a description of the Multimed board itself, as well as the external equipment that is required to use it. To ensure a precise definition of the compatibility between Multimed and other material, both the equipment used in the study and the connectivity requirements are described. By defining the contents and interfaces, this part also sets the boundaries of Multimed’s functionalities.

### 2.1. Position to the State of Practice

The democratization of electrophysiology techniques has motivated the design of many electrophysiology platforms, notably commercial products proposed by National Instruments (NI) [6], Intan [7], Multi Channel Systems (MCS) [8], or open-source alternatives [9], like Neurorighter [10] or Open Ephys [11]. They propose bench-ready, high-performance material and processing software that is capable of real-time processing and feedback. Moreover, the availability of generic acquisition systems, especially from NI, MCS, or Intan gave birth to open-source analysis software [9], including MEABench [12], ArtE [13], Nspike [14], and RTXI [15]. These also provide real-time processing tools and promote always-expanding analysis tools with limited hardware investment.

However, applied research, and particularly, closed-loop research cannot always rely on software processing solutions. Indeed, computation speed is bound by general-purpose operating system performance: processing latencies slower than biological delays limit decision making within physiological windows (e.g., Spike Timing Dependent Plasticity) [16]. Only RTXI claims to achieve consistent sub-millisecond processing, relying on a real-time Linux kernel. On that note, the presence of a workstation in the computation flow disqualifies these setups for implant-scaled miniaturization, and the proposed processing software is too complex for on-chip integration. Some products answer these issues with a hybrid approach, and they take advantage of both software and hardware processing: Intan proposes acquisition devices with a mounted FPGA board that can host low-latency material computing, alongside its standard processing software. A hybrid solution was also proposed in [16], using an FPGA to host a reconfigurable low-latency event engine. It is capable of recognizing spatio-temporal spike patterns and delivering sub-millisecond feedback. Further processing is performed in real-time on computer software, with higher processing latencies. To our knowledge, existing full hardware platforms are dedicated to spike-based [17] or LFP [18] processing and lack versatility to handle wide varieties of activity patterns.

With Multimed, we intend to overcome the limitations of software solutions (lack of integrability and high processing latency) while maintaining the modularity that they offer. It utilizes VHDL (the standardized Hardware Description Language for digital circuits) modules, optimized for resource cost and speed, to assemble full-hardware processing architectures with high performance.

### 2.2. The Multimed Hardware

**Figure 2:**
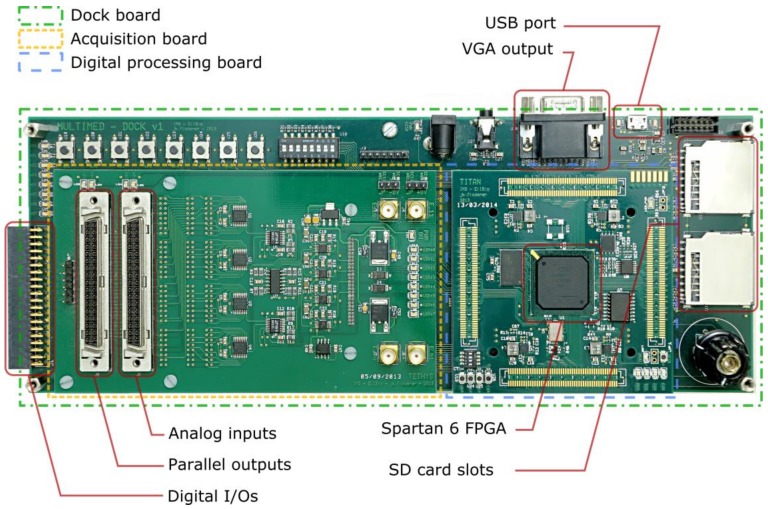
The Multimed system and its hardware interfaces.

The electronics that were used to host Multimed is a custom system designed specifically to perform various operations on multichannel data. It is composed of three custom boards, as shown in Figure 2.

The first board is an acquisition board, acquiring data on 64 channels, at 10 kHz, with 16 bit precision. Optimal use of the ADCs’ input range is ensured using Programmable Gain Amplifiers (PGAs) with gains ranging from 1 to 100. The board has two SCSI68 connectors, which are compatible with Multi Channel Systems 60-electrode equipment. An internal ±8 V power supply can be bridged to pins #1, #67, and #68 (ground, positive, negative) to power preamplifiers. All of the signal pins from the connectors are wired in parallel (leaving the power supply pins independent on both connectors), which means that Multimed can transparently be inserted in an existing acquisition chain: one connector serves as a signal input and the other as a parallel output. Note that the 64 channels are provided for design symmetry reasons; therefore, the four inputs that are unused with the 60-channel connector are accessible via either four SMA connectors or four standard 2.54 mm pitch square connectors.

The second board is both a processing board and a control board. It has numerous I/Os that are connected throughout the system and to its main component, a Xilinx Spartan-6 FPGA. It can access all of the digital components and interfaces in the system, meaning that it can send configuration instructions to any subsystem in Multimed. It also means that it can read the sampled multichannel data from the first board, process it, and send feedback instructions accordingly, all using reconfigurable architectures. To facilitate communication with high-level interfaces, the FPGA embeds a softcore PIC16F-instruction-compatible processor.

The third and last board is an interface board, which includes multiple LEDs, buttons, and switches, but also generic digital I/Os, a 3.5 mm TRS jack output, a VGA output, a micro-B USB port, a JTAG connector, and four SD card slots. It is also the board that distributes all the power supplies throughout the system: both the processing and acquisition board are plugged into and powered by it.

### 2.3. External Equipment

To measure biological signals that are captured by MEAs, ranging in the tens and hundreds of microvolts, Multimed requires an external high-gain, low-noise amplification stage, which is placed as close to the electrodes as possible. In this study, MEA1060 amplifiers by Multi Channel Systems (MEA1060-Inv, MEA1060-Inv-BC, Multi Channel Systems (MCS), Reutlingen, Germany) were used. They can host standard 60-electrode MEAs and have a SCSI68 output connector. They have a gain of 1200 and their bandwidth can be chosen according to the final application (0.1 Hz–3 kHz, 1 Hz–3 kHz, or 10 Hz–3 kHz).

Similarly, Multimed does not have extensive stimulation capabilities and needs stimulation equipment it can trigger. In this study, it has been connected to an STG 4004 by MCS (STG 4004, MCS, Reutlingen, Germany), 4-channel stimulus generator with 4 independent TTL input triggers.

## 3. System Architecture and FPGA Design

Within the context being described in Methods, we designed Multimed, a module-based, multi-application platform dedicated to the acquisition and processing of biological signals. It meets configurability and real-time constraints, and features filtering, detection, measurement, and decision-making capabilities. This section describes the resulting system in more detail. Specifically, it gives an overview of Multimed’s architecture, as well as a list of all the available processing modules. Then, processing performance figures are given. Finally, examples of experiments are given to highlight Multimed’s ability to conduct multichannel recording.

### 3.1. System Architecture

**Figure 3:**
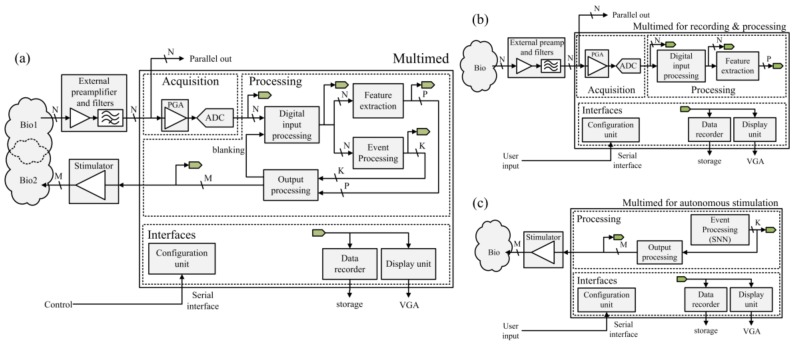
(**a**) Architectural description of the Multimed board and its peripherals. Data acquisition and processing is simultaneous on N channels. P features may be extracted from individual channels or as a synthesis of a cluster of channels. Features and events are processed to control stimulation on *M* configurable channels (trigger/configuration, electrical, or chemical). Stimulation-synchronized digital blanking is available on the *N* recording channels. *N*, *P*, *K*, and *M* are application-specific design parameters. Human-machine interfaces include serial configuration, VGA display, and flash memory (SD card) storage; (**b**) Architecture example for data acquisition and real-time, dynamically configurable processing, recording, and display of biological activity; (**c**) Architecture example for Spiking Neural Network (SNN)-controlled stimulation of a biological medium, where an SNN is used as an autonomous event generator, providing live SNN event recording and display.

The architecture design was primarily motivated by the real-time constraint. This resulted in the implementation of two separate functional groups, as highlighted in Figure 3a, with different aims and different levels of constraint. One group is the processing chain, a sub-architecture responsible for critical real-time signal processing. It steadily performs operations on input signals with fully pipelined or parallelized algorithms. The other is the interface group, which was responsible for managing configuration, recording, and display. It constitutes an environment that controls processing and distributes data between peripherals. It does not take part in signal processing, and consequently, it has a weaker time constraint. This flexibility in latency and refresh rate justifies the use of the softcore processor, which in turn, also facilitates development. The current section describes in detail both functional groups and their interaction.

As represented in Figure 3, signal processing is carried out using a module-based sub-architecture implemented on the FPGA. It performs operations on a generic number of channels, *N*, received from the acquisition board. The first stage of this sub-architecture, *Digital Input Processing*, extracts events from the input data. These events represent the detection of APs, SPs, LFPs, or bursts on a single bit per channel, significantly reducing bitrate. They are either directed into a generic *Event Processing* module with *K* outputs, or a *Feature Extraction* module that calculates *P* event-related features, such as frequencies or synchronization. Finally, the events from *Event Processing* or the extracted features are sent to an *Output Processing* module that formats them into *M* stimulation-driving channels. Commercial multichannel recording and stimulation systems [19] usually contain multiple buffering levels that impede real-time control and feedback capacity. On the contrary, Multimed is fully pipelined or parallelized and provides a constant, well-characterized processing latency. Furthermore, all of the intermediate processing stages are accessible in higher-level layers of the system for both display and recording. All of the processing and display modules are configurable via a computer before and during experiments.

Each Multimed digital processing stage is an application-specific arrangement of processing units from a dedicated library, as detailed in *Processing units* and *Function combinations*. The processing architecture may be remodeled, as illustrated in the examples in Figure 3b,c, depicting, respectively, an open-loop architecture dedicated to signal analysis and monitoring [20] and its counterpart for open-loop stimulation driven by a Spiking Neural Network (SNN). In the latter case, the Central Pattern Generator described in [21] was integrated in *Events Processing* to generate stimulation triggers.

**Figure 4:**
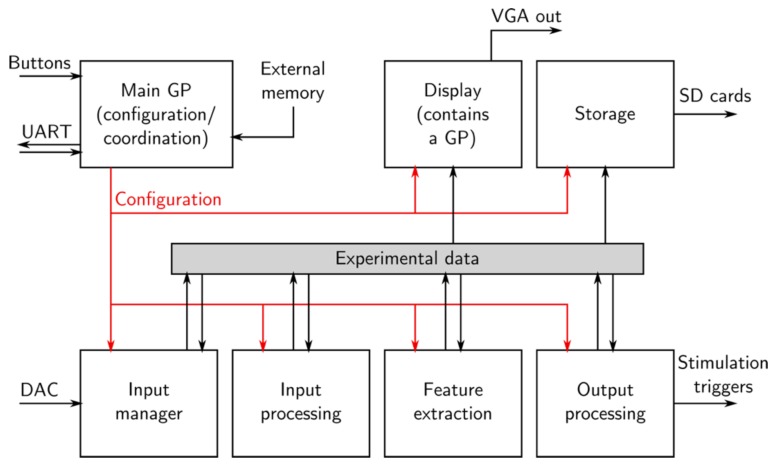
High-level data flow of Multimed. The generic processor (GP) is separate from the data processing chain and only helps as a means of communicating and configuring. All of the digital processing modules feed off the same data bus (Experimental data), minimizing dependencies and facilitating complex connections.

The processing architecture, due to its modularity, requires a flexible environment that will manage the communication between the user interface and the hardware components of the board (later referenced as Main GP). This is where the softcore generic processor (GP) comes into play: it handles all of the operations that are deemed “non-critical” in the real-time domain. These include data recording management and configuration management. The modules responsible for this environment (the Main GP and Storage in Figure 4) can either read experimental data from data buffers or write values in module configuration inputs. However, the resulting read/write operations are designed to have no effect on the signal processing loop timing.

A dedicated softcore environment is also implemented for the display management. Much like the Main GP, this processor is not involved in the real-time closed loop computation, but has access to all data received and processed by the board.

The softcore processor itself controls the system parameters. It is not involved in data processing, but it handles communication between processing modules and hardware peripherals. It interprets user instructions and parameters to distribute them through the system. Available configuration inputs include processing parameters, as well as display and storage unit control. This processor is capable of running either embedded or user software. It also controls the system flash memory, providing user-friendly update capabilities when experiment results raise architecture additions.

Recorded raw and processed data (function outputs) are stored on SDHC cards: 32 GB cards hold up to 7 h of 64-channel raw data sampled at 10 kHz. Data storage is controlled by the processor, but it retrieves data directly from the source, in order to satisfy the real-time requirements. SD storage routines have thus been embedded in the FPGA. Online display is also possible via a VGA port and dedicated graphic processing units have been embedded in the FPGA. This specific module offers a flexible data display, which is entirely configured by the main processor and handled by a specific secondary processor.

### 3.2. Processing Units

As previously stated, Multimed is a multi-application platform with extensive configurability features. For that purpose, and to support interchangeable data flows, a module-based approach has been used, in which communication and processing units are implemented on a FPGA. A library of functional blocks has thus been developed in VHDL, with standardized connectivity rules for easy, straightforward design of real-time, multichannel processing chains.

Processing modules are designed to be fully configurable, with two categories of parameters: dynamic parameters, which are modifiable by the user, while the system is in operation, and static parameters (generally architecture-related, such as the number of channels and data width) set during the architecture design phase. Dynamic parameter implementation has been designed to keep data consistency while updates are performed. The user can then tune parameters while the system is running without risk to record incoherent data.

Data manipulation protocols are also standardized inside and between processing units to maximize compatibility and modularity, as well as optimize the necessary resources per processing channel: event-based processing and transfer are parallelized while digital signal processing and transfer are pipelined. Because our hardware is FPGA-based, there are no pre-allocated resources for a given unit; available hardware resources are only bounded at the system level by FPGA size.

**Figure 5:**
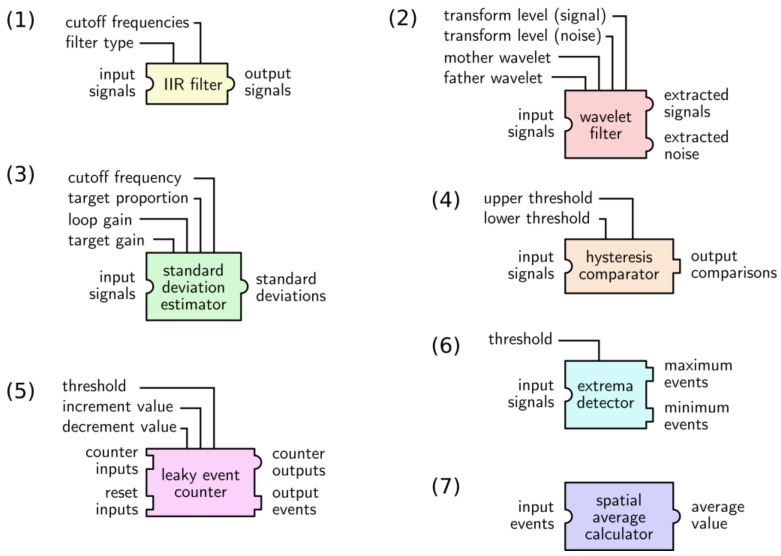
Processing units from Multimed’s module library. Square inputs/outputs represent event connections and round inputs/outputs represent signal stream connections. The numbering of the modules refers to that of both Table 1 and the current section.

This section presents an inventory of the basic processing functions that are available in the VHDL library, as summarized in Table 1 and represented in graphical form in Figure 5, at the end of the section. These elementary functions are assembled in a processing chain for each application. Some commonly used combinations of these functions are described in Section 3.3.

#### 3.2.1. Infinite Impulse Response (IIR) Filters

A Butterworth IIR linear filter module has been implemented to isolate components of interest from biosignals for further processing: for instance, Slow Potential (SP) detection in the 0.2 Hz–2 Hz range [4] requires band-pass filtering before event detection. To optimize memory use and improve configurability, the filter module consists of the eight first-order, low-pass filters in series, with separately configurable cut-off frequencies, $F_{c}$. Each first-order filtering operation is defined by the following equation:

(1a) $$Y_{n}=Y_{n-1}\times \left(1-\frac{1}{2^{k}}\right)+\frac{X_{n}}{2^{k}}$$

(1b) $$\;\frac{1}{F_{c}}=\left(2^{k}-1\right)2\pi T_{s},k\in \left\{0,17\right\}$$

where $Y_{n}$ is the *n*th output sample, $X_{n}$ is the *n*th input sample, and $F_{c}$ is an approximation of the cutoff frequency. Consequently, high-pass filtering is also available by computing the difference between the input signal and the output of a specific portion of the low-pass filter chain. This forms a fully configurable filter unit, which is up to eight orders. Band-pass filtering is achieved by configuring both high- and low-pass filters within the same filter unit.

#### 3.2.2. Wavelet Filters

Consistent spike detection relies on good discrimination of AP waveforms from baseline noise. Though APs may be discerned using only high-pass filtering, a low signal-to-noise ratio will impede detection. We therefore implemented wavelet filters with configurable detail level and mother wavelet coefficients. This enhances AP waveforms that may otherwise be drowned in noise. The SWT (Stationary Wavelet Transform) [22] algorithm is used to compute $d_{n}^{j}$ and $a_{n}^{j}$, the detail and approximation coefficients for detail level *j* at the *n*th sample, respectively:

(2a) $$d_{n}^{j}=\overset{L}{\underset{k=0}{\sum }}g\left[k\right]\times a_{n-2^{j}k}^{j-1}$$

(2b) $$a_{n}^{j}=\overset{L}{\underset{k=0}{\sum }}h\left[k\right]\times a_{n-2^{j}k}^{j-1}$$

where g[k] and *h*[k] are the *k*th coefficient of the high- and low-pass filters, respectively, out of *L* wavelet coefficients. $d_{n}^{j}$ is the useful signal component and $a_{n}^{j}$, the noise component, at the detail level *j*.

#### 3.2.3. Standard Deviation Estimator

APs are detected when the spiking signal exceeds a threshold, which are chosen to establish a clear distinction between samples that constitute noise and those that belong to an AP. However, in multichannel processing, noise levels may vary between channels and over time [5], thus making it impossible to use a single, constant detection threshold.

A commonly used solution in electrophysiology consists of setting the detection threshold to a multiple of the standard deviation of individual channel signals [23]. Therefore, we implemented digital models [22] of analog real-time standard deviation estimators [24]. The chosen model is a closed-loop regulator based on noise distribution hypotheses. It estimates the standard deviation so that a precise proportion of samples exceeds it (15.9% in the case of a white Gaussian noise). In order to accommodate noise-distribution non-idealities, all of the model parameters are accessible for dynamic configuration. Although such a unit is referenced as standard-deviation estimator in the literature, it is in fact used to provide an image of signal amplitude that is rather immune to the cell activity present in the measured signal.

#### 3.2.4. Hysteresis Comparator

A two-threshold asymmetrical comparator is available for all thresholding uses, including event detection and inter-spike interval discrimination. This module output follows a hysteresis cycle that improves noise immunity. The two thresholds are set separately, making it usable as a simple comparator if both thresholds are equal. This comparator is suited for both amplitude and time thresholds.

#### 3.2.5. Leaky Event Counter

Channel activity is quantified by counting the number of events detected. However, in neural applications, the significance of information often decreases with its anteriority, so the leaky event counter, $C_{n}$, gives temporal significance to event quantification by systematically decreasing the event count over time. This “leak” is fine-tuned by changing an increment step, $s_{U}$, and a decrement step, $s_{D}$. The leak value is constant, so this unit actually measures the difference between the instantaneous firing rate and a reference firing rate on a sliding time window. This facilitates the detection of event synchronization and network bursts.

A secondary output of the counter also produces an event when the quantity of activity measured exceeds a threshold, Q. All of the counter features are defined by Equations (3) and (4) below:

(3) $$C_{n}=\{\begin{array}{c} 0,\;\;\;\;C_{n-1}+X_{n}\times s_{U}\geq Q\\ max\;\left(C_{n-1}+X_{n}\times s_{U}-s_{D};0\right),\;\;otherwise \end{array}$$

(4) $$Y_{n}=\{\begin{array}{c} 1,C_{n-1}+X_{n}\times s_{U}\geq Q\\ 0,otherwise \end{array}$$

where $C_{n}$ is the event counter, $X_{n}$ is the input event ,and $Y_{n}$ is the output event, all for sample *n*.

#### 3.2.6. Extrema Detection

Slow Potentials are detected on the basis of local maxima and minima in the low-frequency components of input signals. To avoid the detection of smaller, insignificant ripples, we implemented a module that detects local extrema and discriminates them according to their peak amplitude. An amplitude threshold, Δ, may be configured to define the minimum amplitude between two consecutive extrema. Maxima and minima are detected separately and alternately. When the module expects a maximum, the detection occurs, according to the following criteria:

(5a) $$M_{n}=\{\begin{array}{c} X_{n},\;\;X_{n}>M_{n-1}\\ X_{n},\;X_{n}\geq m_{n-1}+\Delta \\ M_{n-1},\mathrm{otherwise} \end{array}$$

(5b) $$Y_{n}^{M}=\{\begin{array}{c} 1,\;X_{n}\leq M_{n-1}-\Delta \\ 0,\mathrm{otherwise} \end{array}$$

where $Y_{n}^{M}$ is the *n*th output sample of the maximum detection and $X_{n}$ is the *n*th input sample. The maximum value of *X* up to sample *n* is stored in $M_{n}$, and is considered a maximum only when the signal drops below this value by at least Δ, at which point $M_{n}$ is reset and the module enters a minimum detection mode, which operates similarly:

(6a) $$m_{n}=\{\begin{array}{c} X_{n},\;\;X_{n}<m_{n-1}\\ X_{n},\;X_{n}\leq M_{n-1}-\Delta \\ m_{n-1},\;\mathrm{otherwise} \end{array}$$

(6b) $$Y_{n}^{m}=\{\begin{array}{c} 1,\;X_{n}\geq m_{n-1}+\Delta \\ 0,\mathrm{otherwise} \end{array}$$

where $Y_{n}^{m}$ and $m_{n}$ are the counterparts for $Y_{n}^{M}$ and $M_{n}$, respectively, whenever the module expects a minimum.

#### 3.2.7. Spatial Average Calculator

It is possible to integrate the measurements from several channels by computing their instantaneous mean: the spatial average calculator module averages any combination of *N* streams into one. The operation is optimized for speed as it is bound to be repeatedly performed at the sampling frequency.

A decomposition of the averaging operation is first computed using Equation (7), which is performed once as a Euclidian division:

(7) $$A=2^{W}/N$$

where *W* is the data width. Let *J* be the set of *N* channel data to be averaged. The spatial average *Y* is computed by Equation (8), where $a_{i}$ is the *i*th digit of the binary representation of A:

(8) $$Y_{n}=\overset{W-1}{\underset{i=0}{\sum }}a_{i}\times \frac{\sum_{j\in J}X_{n}^{j}}{2^{i+1}}$$

where $Y_{n}$ is the *n*th output sample and $X_{n}^{j}$ is the *n*th input sample for channel *j* within *J*.

### 3.3. Function Combinations

**Figure 6:**
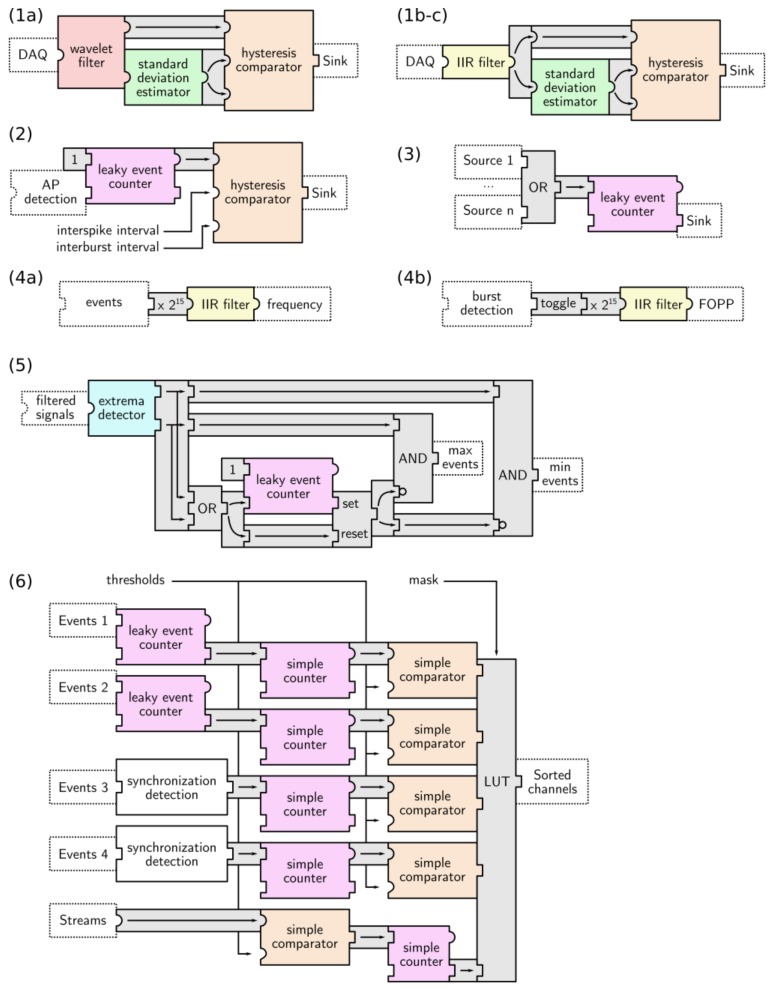
Combinations of processing units generate more complex functions. Numbering refers to that of Table 2. (**1a**): Adaptive wavelet AP detection; (**1b-c**): Adaptive AP-Local Field Potentials (LFP) detection; (**2**): burst detection/AP cleaning; (**3**): Synchronization detection; (**4a**): Event frequency measurement; (**4b**): Burst Fraction of Plateau Phase (FOPP) measurement; (**5**): Extremum detection; and, (**6**): Electrode sorting. Blocks in dotted lines indicate connection context.

The processing functions described above are commonly used in isolation (e.g., isolating AP waveforms may only require one IIR filter bank), but they can be assembled into more complex processing units (e.g., adaptive AP detection requires a combination of a filter bank, threshold computation with a standard deviation estimator, and a threshold comparator). To make complex architecture design easier, such combinations have been made available as building blocks, as listed in Table 2 and detailed in Figure 6. In order to maintain full configurability, all of the parameters from the sub-blocks (listed in Table 1) are accessible in these functions.

#### 3.3.1. AP/LFP Detection

One of the most commonly used detection functions is AP detection. Two variants are available in the VHDL function library, according to the type of AP. For clearly discernible APs (e.g., in patterned neural cultures), the detection module consists of a band-pass filter to isolate waveforms of interest, a standard-deviation estimator for computing the detection threshold, and a simple comparator to determine when the spiking activity exceeds the threshold. If the APs cannot be clearly distinguished from the baseline noise, a variant module includes wavelet filters instead of linear filters [22]. Note that fixed threshold detection may also be implemented while using a simple comparator module.

Similar to APs, LFPs are commonly detected by filtering (at frequencies typically two orders of magnitude below those of APs) and thresholding. The same module is used for detection, but wavelet filters are not used as LFP amplitude generally provides good discrimination.

#### 3.3.2. Burst Detection

Bursts of activity are detected using a leaky event counter and a hysteresis comparator. When comparing the event count to a threshold generates the burst event while hysteresis provides different time sensitivities in detecting the start and end of a burst. The burst detection module also incorporates a spike cleaning function: all successful AP detections are followed by a configurable period, during which all of the incoming events are blanked. This reduces noise-induced detection artefacts.

#### 3.3.3. Synchronization Detection

Synchronization detection identifies network bursts in patterned cultures, or faulty electrodes that are electrically coupled, while using a leaky event counter. Events from different channels are mixed and increment the leaky event counter. The value memorized by this counter thus represents a quantification of recent network activity. If this quantity of activity exceeds a threshold, a synchronization event is flagged.

#### 3.3.4. Event Frequency and Burst Fraction of Plateau Phase (FOPP)

Event frequency and burst FOPP are representative of channel activity. They are computed using IIR low-pass filters with a configurable time constant and order (first or second). The module acts as an averager: of the burst envelope in the case of the FOPP and single events in event frequency computation.

#### 3.3.5. Channel Sorting

Once individual channels have been processed, it is relevant to increase their statistical significance by processing sets of channels. An electrode sorting module, as described in [25], is used to select the channels of interest. Four sorting banks separately sort channels according to user-defined criteria, including:

- Raw signal amplitude exceeds a threshold value.
- Occurrences of a given event (AP, SP, or LFP) exceed a threshold.
- Channel synchronization has been detected (one or more times). In this case, all of the channels that contributed to the synchronization are flagged.
- This first stage yields four intermediate sorting results. After that, a user-defined logic Equation defines the final sorting results by combining the results from every bank.

Automatic sorting criteria are computed either on request or at regular intervals in time. The actual sorting is, however, only updated at the end of a measurement period. The set of sorted electrodes can, for example, feed the spatial averager.

#### 3.3.6. Stimulation Control

The stimulation control module provides an appropriate trigger to the stimulator or stimulation source: a proprietary integrated circuit or instrument. We compiled a library of stimulator-dependent modules. A module control output may be as simple as a TTL line to trigger an externally-preconfigured stimulator, or a higher-level communication protocol that both modulates and triggers a stimulation event.

TTL-triggered stimulators have the lowest response time, while the modulated stimulators are more flexible for complex feedback in closed-loop configurations. Although stimulation control is provided by our module in a very short time (under 10 µs), the time cost of the full stimulation and the decision-making scheme is experiment-dependent and may be in milliseconds, depending on the number of required samples for decision-making.

**Figure 7:**
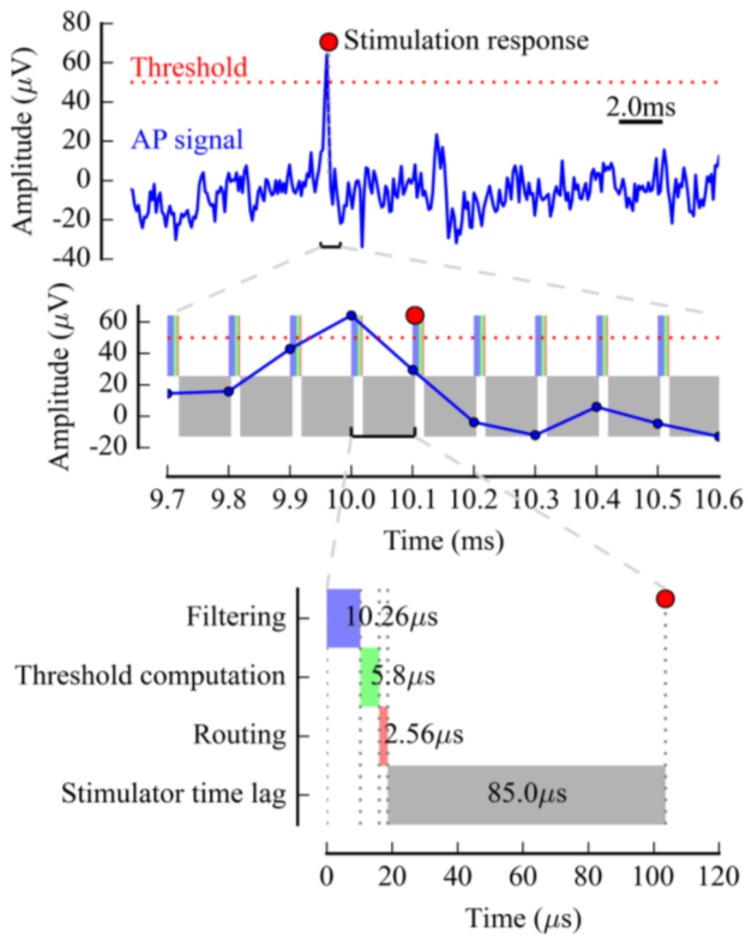
Timing example of a feedback stimulation chain in a neuron culture. Simple spike detection was used with linear filtering, adaptive threshold, and direct routing. The colored time intervals represent the system computation lag. The stimulator time lag, represented in gray, is also taken into account. The time lag shown is that of the MCS STG 4004 Stimulation Generator (STG 4004, MCS, Reutlingen, Germany), which was previously used with the system. The “stimulation response” mark represents the instant at which the electrical stimulus was sent.

To illustrate stimulation generation performance in Multimed, Figure 7 presents the computation steps in a simple case: in a 64-channel acquisition chain, a single AP is detected by IIR filtering and adaptive thresholding, and triggers a stimulus on an MCS STG 4004 (STG 4004, MCS, Multi Channel Systems, Reutlingen, Germany). All of the processing steps between the raw signal and the actual electrical stimulus (filtering, threshold computation, and routing) are represented, including the external stimulator time lag, as specified by the manufacturer. In this case, our stimulation control module output a TTL trigger and computed the “routing” step in 2.56 µs, giving a total of 103.62 µs between the AP and the stimulus.

The stimulation control is associated with a blanking function that drops any event detected within a time window after the stimulation has been triggered. Blanked channels and window time are configurable (in the 0–25 ms range per 0.1 ms step for blanking time) and are closely related to the stimulation scheme. Blanking is necessary to reject stimulation artifacts, otherwise considered as intrinsic cell activity, thus making the measurements irrelevant.

Commercial preamplifiers, such as MCS’ MEA1060-Inv-BC (MEA1060-Inv-BC, MCS, Reutlingen, Germany), include a built-in analog blanking circuit. Multimed’s digital blanking, in contrast to analog blanking, only cancels event detection and processing, thus maintaining continuous acquisition during stimulation artifacts.

### 3.4. Performance

Measurements have been performed to validate every available function. However, with processing and detection accuracy being highly dependent on the nature of the signal and the processing parameters, validation was mainly algorithm-driven; proper algorithmic operation was verified and visual controls validated the accurate detection of events on neural and pancreatic biosignals during experiments. An in-depth validation of wavelet adaptive detection of APs is detailed in [22].

The main advantage of the Multimed system is the small amount of hardware resources it requires for the functions it provides. Computation is performed in fixed point representation with a specific bit-length precision and uses simple, resource-efficient computation schemes, which is unachievable using generic processors or DSPs. The dedicated architecture also makes a high number of operations per clock cycle, thus improving the efficiency of the whole system. Our VHDL modules also receive generic parameters that remove unwanted channel computation. This feature results in an excellent time- and resource-cost per acquisition channel (as detailed in Table 1 and Table 2), regardless of the number of channels.

All of the control and interface functions share the same FPGA as the data processing functions, thus limiting the available resources. In a standard configuration, control functions use 10% to 40% of RAM resources (the worst case is due to the memory buffer necessary for SDHC cards) and 15% of distributed logic.

To improve performance, data is transferred between modules in a flow. At the module level, *N* samples corresponding to *N* electrodes are serially input and output. Simple modules are fully pipelined and implemented at very little resource and time cost, while more complex modules are not fully pipelined: data is buffered in a local memory. Although this communication scheme may sometimes induce duplicated storage in the system, it improves the processing latency (quantified in Table 2).

Multimed features are summarized in Table 3. With the described environment, it is relatively straightforward to build a fully real-time system where maximum processing latency remains well below the sampling period. Furthermore, the user can freely upscale full data processing chains, even if the overall processing latency is longer than the sampling period, as all of the computation modules obey the real-time processing constraint (“real-time processing constraint” meaning that computation time is bounded by design). Processing latency remains constant, with no necessity for data buffering, and no risk for data overflow. This is an advantage for long-term (chronic) experiments that require a high sampling frequency and complex processing, on the one hand, but provide long physiological responses, on the other hand. Multimed’s modest computation resources and high efficiency result in an architecture ready for battery-powered devices with a small form factor. Associated with its autonomy in stimulation decision, it is ready for the design of embedded, closed-loop healthcare architectures.

## 4. Existing Applications

This section presents a series of experiments utilizing Multimed in different application fields. To illustrate its capabilities, a relevant experimental result is given for each application, alongside the corresponding system architecture. All of the experimental procedures were approved by the French Ministry of Education and Research.

### 4.1. Biological Protocols

In neuron preparations, cell activity was measured using 60-electrode MEAs from the Qwane Biosciences Company [Qwane, Lausanne, Switzerland]. These biochips provide 60 platinum electrodes with 40 μm-diameter tips and 200 μm spacing, a larger area for the grounded electrode, and a 6 mm-high glass cylinder, used as a culture chamber. Primary neuron cell cultures were obtained from the cortex of embryonic (E18) Sprague-Dawley rats, following a standard protocol (see [26] for a full protocol description). The MCS pre-amplifier (MEA1060-Inv, MCS, Multi Channel Systems, Reutlingen, Germany) had a gain of 1200 and raw data were sampled at 10 kHz/channel. Neuron culture age was 48 DIV (Days in Vitro) in the monitoring application and 36 DIV in the stimulation application; both when neuronal activity mainly consisted of bursts [27,28].

In pancreatic islet preparations, activity was measured using 60-electrode MEAs in two configurations, including one where drug concentrations could be continuously modulated by using microfluidic circuitry. These were custom MEAs, supplied by Qwane Biosciences company (Qwane Biosciences, Lausanne, Switzerland), featuring 30 Platinum electrodes with 30 µm-diameter and 150 µm spacing. Other MEAs, as obtained from MCS [MEA200/30-Ti-gr from Multi Channel Systems, Reutlingen, Germany], featured 60 Titanium nitride (TiN) electrodes with 30 µm-diameter and 200 µm spacing, together with a 6 mm-high glass cylinder used as a culture chamber. Whole murine islets were cultured for 2–7 days on the MEAs, as described in [4,20]. The pre-amplifier was a MCS 60-electrode amplifier (MEA1060-Inv-BC from Multi Channel Systems, Reutlingen, Germany), custom with a gain of 1100 and a bandwidth of 0.1–3000 Hz.

### 4.2. Experiment #1: Recording and Detection

**Figure 8:**
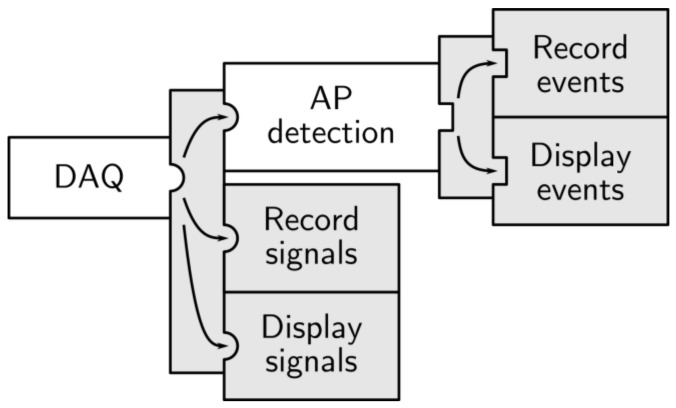
Multimed processing architecture for the detection and recording of neural spiking activity.

**Figure 9:**
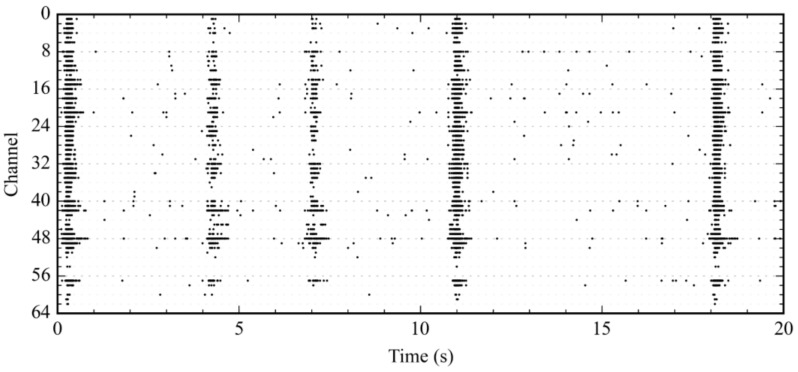
Raster plot of real-time neural AP detection on a patterned culture, recorded on a 60-electrode MEA. In this reading, the average burst contained 11 APs per electrode. The experimenter validated detected events offline.

**Figure 10:**
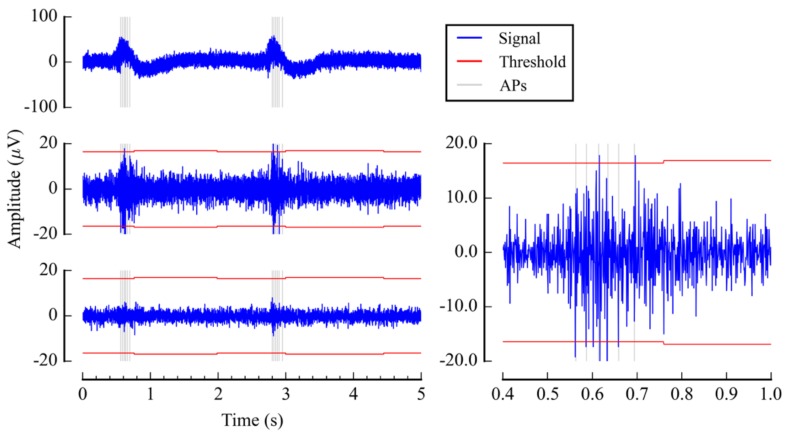
Detail of real-time AP detection on a neuron culture using an adaptive threshold and Haar wavelet filtering. Signals shown are: A: raw signal and AP detection; B: 4th wavelet transform level on which detection was performed, detection threshold, AP detection; and, C: 1st wavelet transform level, on which the detection threshold was computed, detection threshold, and AP detection. The experimenter visually validated detected events.

Neuronal response to mobile telephony signal exposure was investigated by monitoring the electrical activity of neural cultures on MEAs over long periods of time. The study, as published in [29], revealed a reversible decrease in firing and bursting rate during exposure to a 900 MHz mobile telephony signal (900-GSM). The Multimed architecture used for these experiments was a direct processing chain, with no feedback (see Figure 8). The 60 channels were processed, stored, and displayed in real time. Digital processing included AP, LFP, and burst detection. Figure 9 displays a raster plot of adaptive wavelet AP detection. Low amplitude (tens of microvolts) APs were discriminated using Haar wavelet filtering and adaptive thresholding, as illustrated in Figure 10. Wavelet parameters were defined according to validated data for AP detection on rodent cortical neurons [22].

This series of experiments, conducted in parallel with commercial hardware, helped to validate the ability of the system to render real-time analyses of spiking activity in neuron cultures with an open-loop architecture.

### 4.3. Experiment #2: Screening and Processing

The process of testing new drugs in pharmacology involves subjecting live cells to chemical compounds and measuring a range of parameters to assess the quality of the drug. Those parameters reflect the way that the cells reacted and helped to deduct which of their mechanisms were altered. The Multimed platform introduces an electrophysiological approach to this process, displaying the effects of the drug in real-time. This approach has the advantage of providing time-dependent information on the effects of the drugs, while eliminating the need to take samples from the preparations being tested.

The electrical activity of pancreatic islets is an integrative read-out that encodes the body’s demand for insulin. Various techniques (thermal or impedance spectroscopy, fluorescence, photoacoustic, or electromagnetic sensing, etc.) are available to investigate the effects of new drugs in the treatment of diabetes, while our system provides direct access to the physiological response of the islets [4]. Screening measurements were conducted to determine the behavior of murine pancreatic islets in response to changes in the main physiological stimulus (Glucose) and can be easily extended to other nutrients and hormones.

**Figure 11:**
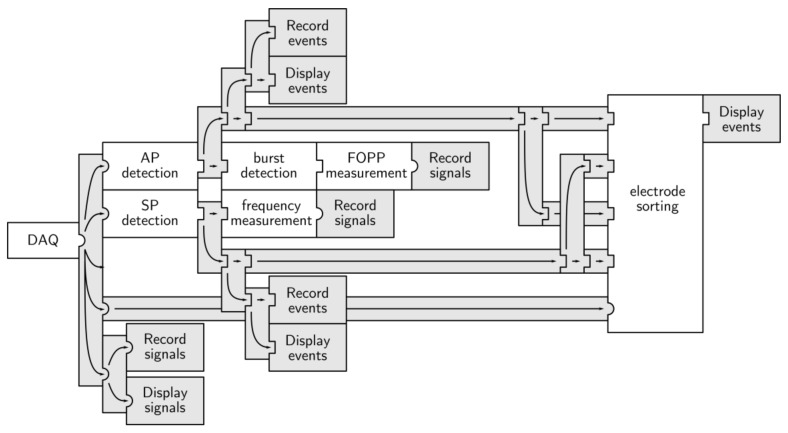
Multimed processing architecture for pancreatic islet screening.

**Figure 12:**
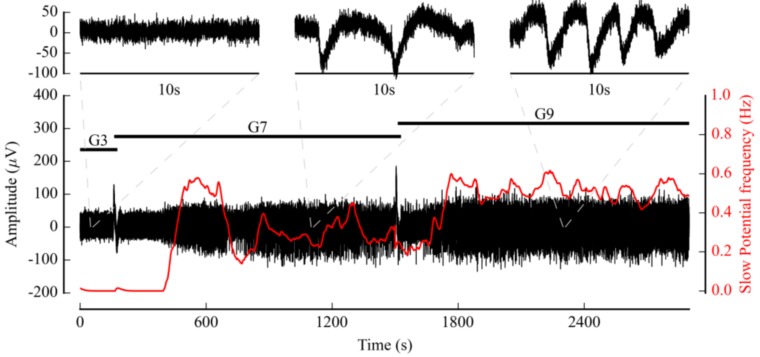
Raw signal (black) and Slow Potential (SPs) frequency measurements (red) on a mouse islet culture, using a Qwane MEA with microfluidic features. Zoom on three representative samples of the measured biosignal to provide a clearer illustration of changes in measured activity. Experimental protocol is described with the “G3”, “G7”, and “G9” labels, indicating changes in glucose concentrations in mM/L.

The Multimed configuration (see Figure 11) for this application was able to record raw data, as well as detecting APs, AP bursts, and Slow Potentials (SPs). It also included measurement modules to compute AP and SP frequency and FOPP, as well as the spatial average of those parameters. Figure 12 shows the changes in both signal amplitude and SP frequency in response to changes in the perfused glucose solutions. Real-time processing emphasizes the dynamics of islet response, particularly when the glucose concentration increased (from G3 to G7).

**Figure 13:**
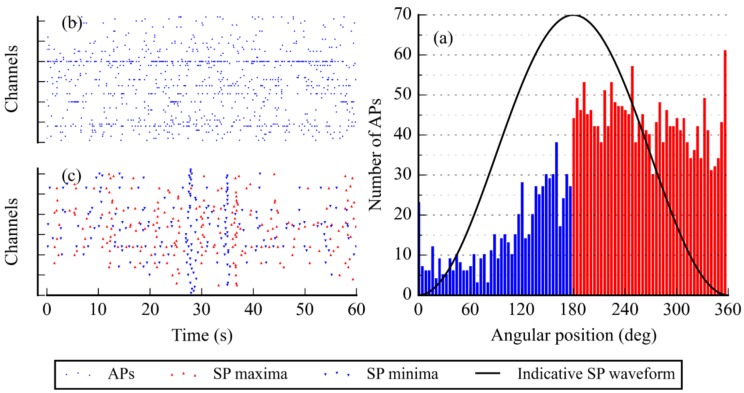
(**a**) Histogram of the angular position of APs on SPs, detected in real-time. Classes are 4°-wide and sort 2608 APs from 329 SPs; and, (**b**,**c**) Sample raster plots of AP and SP real-time detection, used as a base for the histogram. The recordings used lasted 300 s in total.

It is also possible to perform statistical analyses, and more specific, costly calculations on events. We hypothesized that SPs were better biomarkers of islet function and insulin demand than APs [4]. In [4], we validated the AP-SP correlation by detecting APs and SPs off-line and evaluating their respective angular position. We reproduced that result using Multimed’s online and real-time detection. Figure 13 shows an example of a statistical analysis that was performed on a set of online-recorded data on mouse islets (MCS 60-channel MEA). 2608 APs and 329 SPs were detected in real-time and the computation of their relative positions, as performed in MATLAB (MathWorks, Cambridge, UK), revealed that the majority of APs fired during the downward phase of SPs. Though the system does not perform such analyses entirely in real-time, real time signal pre-processing significantly reduces the transfer and post-processing time.

### 4.4. Experiment #3: Towards Closing the Loop

Biosignals recorded in vivo present signatures that were identified as representative of a physiological state, and hence, relevant for physicians, and suitable for controlling feedback stimulation to restore a healthy state: beta-band enhancement is a marker for motor symptoms in Parkinson’s disease [30]; Slow Potentials act as glycaemia markers for diabetes control [4]; responsive cortical stimulation is acknowledged as an efficient epilepsy treatment [31]; and, cortical spike patterns are used to control prosthetics limbs [32]. Signal features may also reflect physiological changes that require a modification of the stimulation parameters. With that in mind, designers refer to real-time control systems as closed-loop control systems where one has a tight time window to gather data, process that data, and update the system.

**Figure 14:**
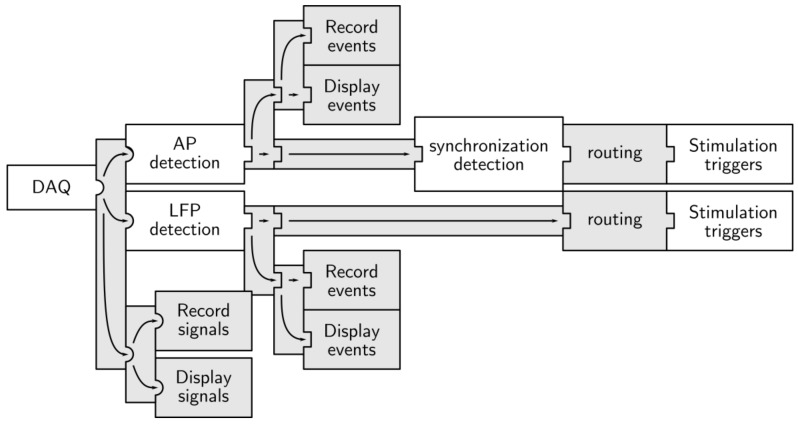
Architecture for the closed-loop stimulation of neuron cultures.

Multimed’s performance in decision-making and its architectural possibilities were designed to make it suitable for closed-loop experiments involving pulsed stimulation. It would act as a feedback controller for an external stimulator that loops back into the biological medium. The main advantage when compared to previously presented architectures is the *Output processing* module, which configures different signals, combinations of signals, and network dynamics as the source of one of the stimulation trigger outputs (see Figure 14).

**Figure 15:**
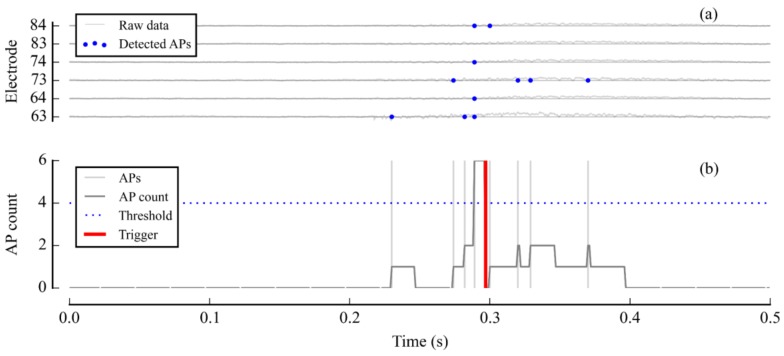
A stimulation command triggered by synchronized spiking activity in a neuron culture. The trigger was set off when more than four APs were detected across six selected channels in a 25 ms time window. (**a**) Unprocessed data on one of the channels of interest; (**b**) AP detection on the six channels of interest and, (**c**) Illustration of synchronization detection: AP count and related trigger.

Figure 15 presents the results of a demonstration experiment on a cortical neuron network, in which a stimulation trigger was sent whenever the system detected network synchronization between six user-defined channels.

In this experiment, no stimulation artifact was observed as no stimulation was actually performed on the culture. Closed-loop experiments using Multimed were recently performed in two research projects: the BRAINBOW project [33], exploring functional organization and the dynamics of damaged parts of the central nervous system, and the CENAVEX project [34,35], which implements a real-time control scheme for ventilation after spinal cord injury impairing respiratory functions.

With these experiments, we illustrated different uses and configurations of Multimed to showcase its features. First, event detection was shown in a monitoring-only configuration utilizing mouse neuron cultures. Then, frequency measurements were presented to illustrate variations in pancreatic Slow Potentials in mice. It was suggested that online results could be exploited immediately off the SD cards, but that they could also serve as pre-processed signals that cuts-off computation time in otherwise computationally-heavy and time-consuming analyses. It was finally hinted that routing in Multimed had been designed to permit closed-loop experiments, and that single events, as well as network bursting, could be utilized as stimulation triggers. These results demonstrated the viability of a system such as Multimed, as it could successfully adapt to the various materials and paradigms proposed. As a sidenote, we remind that the performance of each submodule remained constant, even with the increasingly complex architectures presented. This again underlines the advantage in utilizing distributed logic, rather than software processing.

## 5. Discussion

Few recording systems described in the literature perform multi-application, multichannel, real-time processing with closed-loop and versatility constraints. The Neurorighter [36] open-source platform meets those constraints and is a great example of a versatile system: all processing is performed in computer software, which makes it extremely flexible. This, however, is to the detriment of global latency and integrability. Software solutions generally suffer from these drawbacks, though latency can greatly be reduced with dedicated RTOS (real-time operating systems), like is the case with RTXI and its 60 µs closed-loop latency [15].

A more comparable system is that depicted in [16], involving a closed-loop neural network consisting of cortical neuron clusters interconnected with electronic synapses. Synapse-based stimulation is computed on a reconfigurable event processor: in order to minimize processing latency and to achieve acausal synaptic stimulation and decision making within the STDP window, critical processing is done in an FPGA. The system’s processing architecture in itself is not configurable, but the decision-making event engine is extremely flexible. Although Multimed features a similar module, the one that is depicted in [16] is more complex and capable of creating entire event-processing sub-architectures.

Comparable hardware is produced by Intan (Intan Technologies, Los Angeles, CA, USA) with its RHD2000 interface board product line: an acquisition board is mounted with an Opal Kelly FPGA board and has SPI interfaces to connect various Intan headstages. This hardware, with its open-source control and real-time processing software, is the product of choice for recent open-source electrophysiology projects. While the community recognizes the potential of Intan hardware to perform sub-millisecond feedback thanks to its FPGA, it is not a native feature and no dedicated modules have been released yet.

Finally, Open Ephys proposes a very popular system that is targeted at researchers that uses implantable devices in non-human subjects. It is an open-source system that can be built at a low price (less than $100 per channel). Although its electronics utilize an FPGA, signal processing is performed in software, resulting in fairly high processing latencies: the closed-loop latency (typically between 10 and 20 ms) is attributed to software buffers and USB communication delays [11]. Again, this project trades a great deal of flexibility for performance, as all data is available for online display in software.

With Multimed, we hope to bring out the best of both worlds, with the performance of full-hardware processing and easily reconfigurable modules and architectures. Still, Multimed’s most immediate limitation is its current analog front-end that limits its usage to 60 electrodes. Sampling frequency is less constrained: the 10 kHz sampling frequency that we used in experiments is set to keep coherency with the 3 kHz bandpass of our pre-amplifiers; that sampling frequency can be increased to 15 kHz for all of the channels without any modification to Multimed. The individual channel sampling rate can also be increased simply by reducing the number of recorded channels to maintain the global sample rate to its current value.

The Multimed computing architecture would not be immediately impacted by higher input figures (e.g., number of electrodes or frequency), as processing modules are parameterized and scalable. Still, implementing the modules library to process more inputs may at some point require a FPGA upgrade to provide sufficient hardware computation resources: Multimed is now equipped with a lowcost Spartan 6 FPGA (Xilinx xc6slx150), which is used at 50% of its computation capacity for the whole system. We performed a theoretical study that showed that same FPGA can handle in Multimed more than 1024 individual channels, the limiting resource being embedded memory.

Upscaling input data (number of electrodes or sampling frequency) will also multiply the amount of data to save in the same proportion. To be able to keep up with this extra amount of data, the system would need extra storage capability. Final storage is not the primary issue, since SD-XC cards can now handle eight times more data that our reference 32 GB SD-HC card, which corresponds to 14-h recording of 60 electrodes sampled at 40 kHz. The main issue comes from the SD-XC pre-record data buffer: its required size has not yet been fully characterized by the authors, when embedded in the Multimed control architecture.

It is also worth mentioning that using highly-configurable systems, such as Multimed, requires appropriate training for end-users: biologists or clinicians. Although Multimed modules are designed to be easily interconnected through a standardized interface, building a customized architecture requires experimenters that are trained in digital system design. Overcoming this constraint would require an advanced, high-level, human-machine interface, which is suitable for use in constrained environments. This interface has not yet been developed, since it was not a functional priority.

The Multimed system can be considered either as an electrophysiology platform or as a prototype for electroceutic devices, such as cortical stimulators, which now bundle many separate functions into a single implantable package [37,38]. Specifically, the synthesized digital architecture provides a ready-to-use netlist for on-chip physical implementations. Multimed settles a proof of concept, but more development is required to produce an embeddable device. A VLSI implementation would greatly improve package size and power consumption, but the resulting IC would be highly application-dependent.

Multimed’s architecture brings massive multi-electrode computing and multi-source fusion within reach, but few experiment paradigms to date have required such performance levels. For that matter, high multidisciplinary collaboration is crucial to define processing parameters and resource-efficient algorithms, while working with signals with partially unknown features.

## 6. Conclusions

Our architecture demonstrated its accuracy in real-time, long-term [4,21,33,34,35,39] experiments. Furthermore, current FPGA use is below its maximum processing potential, as only a few electrodes and simple feedback rules have been exploited. The system that is presented here represents technological advancement in the field of open- and closed-loop experiments for fundamental research in bio-mechanism exploration and functional rehabilitation. It consists of a highly-configurable, generic hardware, which is associated with a library of interoperable modules. The resulting platform makes it easy to build application-specific, open- or closed-loop setups, with high computing efficiency and a large number of channels. Targeting the use of living cells as sensing elements, Multimed provides long term, continuous monitoring, and/or processing of signals recorded by bio-sensors. As recording and display are only used for characterization and evaluation, removing these modules produces an autonomous, embeddable system. This device is then able to perform multi-parametric, multi-channel monitoring and analysis, and provide condensed reports, consultable from time to time (e.g., once or twice a day), via a low-bandwidth connection.

Multimed setups have been installed in multiple sites and are being used by partner laboratories in collaborative projects, always aiming for closed-loop experiments (BRAINBOW (EU project 284772, ICT- FET FP7/2007–2013, FET Young Explorers) [33], CENAVEX (ANR grant 2013-NEUC-0001-01 and NIH grant 5 R01 NS086088-02) [34,35,40], ISLET CHIP (ANR grant 2013-PRTS-0017) [25,41], HYRENE (ANR grant 2010-BLANC-0316-01) [21]). New needs in terms of processing capabilities arise from existing collaborations and can also emerge from new collaborations, leading to regular updates of the digital library. In parallel, hardware developments are ongoing, in an effort to improve the interfaces with our custom high-voltage stimulation ASIC and facilitate the prototyping of fully-customized, autonomous System-in-Package solutions. We also consider interfacing Multimed with commercial sensor modules to investigate new applications that are based on alternative modalities, such as temperature or protein sensing.

## Figures and Tables

**Table 1:** List of the elementary real-time processing functions.

#	Elementary Function	Processing Latency (Clock Cycles)
A1	Linear filters	2*N k* + 2 *
A2	Wavelet filters	From 7*N* to 65*N* **
A3	Standard deviation estimator	9N+4
A4	Hysteresis comparator	4N
A5	Leaky event counter	N
A6	Extrema detection	6N+1
A7	Spatial average calculator	N+W+1

* *k* filters; ** mother wavelet-dependent; *W*: data width; *N*: number of channels. The numbering in the left-hand column is used for further reference. Notations are coherent with those of the equations in part III: *N* is the number of channels and *W* the data width.

**Table 2:** Combined functions.

#	Combined Function	Elementary Parts Used	Dependencies	Processing Latency (Clock Cycles/Channel)	Memory Cost (Bits)
B1a	Adaptive wavelet AP Detection ^1^	A2 + A3 + A4		From 16N + 4 to 74*N* + 4	From 256*N* to 16,512*N*
B1b	Adaptive AP Detection ^1^	A1 + A3 + A4		16N + 6	512*N*
B1c	LFP detection ^1^	A1 + A3 + A4		16N + 6	512*N*
B2	Burst detection/AP cleaning ^1^	A5 + A4	B1a or B1b	4N	$128N\times 2^{16}$
B3	Synchronization detection *^2^	A5		4*N* + 1	8*N*
B4a	Event frequency computation ^2^	A1	B2	9	72*N*
B4b	FOPP computation ^2^	A1	B2	9	72*N*
B5	Slow Potential detection ^1^	A6 + A4 + A5	A1	7N + 1	48*N*
B6	Channel sorting *^2^	A5 + A4 + B3		9*N* + 3	152*N*
B7a	TTL stimulation control (MCS) *^3^	Any event source		stimulation dependent	14 + 4*S*
B7b	High level stimulation control (CNE) ^3^	processor	B6a	stimulator dependent	stimulator dependent

* Processing on multiple channels; ^1^ part of the *digital input processing* unit; ^2^ part of the *feature extraction* unit; ^3^ part of the *output processing* unit; Notations are consistent with those of the Equation (1) through Equation (8): *N* is the number of channels, and *S* the number of stimulation channels. Assemblies are detailed in Figure 6.

**Table 3:** Full system features.

Number of independent analog inputs	64
Analog input/output ports	2×SCSI68 *
Number of generic digital I/Os	10
Amplifier stage gain	×0-1-2-5-10-20-50-100
Bit depth	16 bits fixed point
Sampling frequency	10 kHz
Raw data generated	73.24 MB/min
Maximum recording time	7 h
Display output port	VGA
Communication port	micro-B USB
Communication protocol	Serial, 115,200 baud

* 60 channels, MCS-compatible.

## Data Availability

Please contact author for data requests.
